# Navigating Hepatitis C care: Knowledge gaps and access barriers among young women who inject drugs in rural Appalachia

**DOI:** 10.1371/journal.pone.0331849

**Published:** 2025-09-09

**Authors:** Cheyenne R. Wagi, Renee McDowell, Anyssa Wright, Kathleen L. Egan, Christina S. Meade, April M. Young, Madison N. Enderle, Angela T. Estadt, Kathryn E. Lancaster

**Affiliations:** 1 Wake Forest University School of Medicine, Department of Implementation Science, Winston-Salem, North Carolina, United States of America; 2 Ohio State University, Columbus, Ohio, United States of America; 3 Wake Forest University School of Medicine, Department of Translational Neuroscience, Winston-Salem, North Carolina, United States of America; 4 University of Kentucky, Department of Epidemiology and Environmental Health, Lexington, Kentucky, United States of America; 5 Ohio State University, College of Public Health, Division of Epidemiology, Columbus, Ohio, United States of America; Centers for Disease Control and Prevention, UNITED STATES OF AMERICA

## Abstract

**Background:**

Hepatitis C virus (HCV) and injection drug use among young women are dramatically rising in the rural United States. From 2004 to 2017, heroin use among non-pregnant women increased 22.4% biennially, mirroring increases in HCV cases, especially among younger populations. Young women who inject drugs (YWID, ages 18–35) face elevated HCV risk due to biological, behavioral, and socio-cultural factors. Barriers to HCV testing and treatment services further delay diagnoses, fuel transmission, and limit access to harm reduction services. This study applies the Theoretical Domains Framework (TDF) to identify factors influencing HCV testing and treatment among YWID in rural Appalachia Ohio.

**Methods:**

We conducted in-depth interviews with YWID (n = 30) in 2023 to understand their HCV testing and treatment experiences in rural Appalachia Ohio. Interviews were transcribed, inductively coded, and analyzed using grounded theory. Identified themes were mapped onto the TDF domains.

**Results:**

Key TDF domains influencing HCV care included knowledge, beliefs about consequences, and intentions. While YWID knew where to get tested, they expressed uncertainty about treatment value and access while actively using drugs. Social influences, stigma, and mistreatment by healthcare providers created barriers to treatment. Environmental context and resources, such as transportation, also influenced access to care.

**Conclusions:**

YWID in rural Appalachia face barriers to HCV care, such as gaps in knowledge about HCV treatment, which is compounded by gendered stigma, and logistical challenges. Rapidly changing treatment restrictions led to misinformation about treatment access. These gaps highlight the need for interventions specifically designed to address YWID lived experiences.

## Background

Hepatitis C virus (HCV) and injection drug use (IDU) among young women are dramatically rising in the rural United States [1,2]. Between 2002–2013, heroin use among women increased by 100%, double that of their male counterparts [3,4]. This alarming trend is paralleled by an increase in HCV cases, especially among younger populations during the same period [1]. Acute HCV infections in 2022 were reported at a two times the rate of 2015 and people aged 30–39 represented in the population with the highest rates of acute HCV [5]. Within the population of young people, young women face nearly double the risk of acquiring HCV compared to their male counterparts, placing them in a risk category higher than older women or men within their age category, and this disparity is particularly pronounced in rural regions such as Appalachia [6]. HCV rates among reproductive-aged women (15–44 years) doubled between 2006 and 2014 [7]. Rural areas, including Appalachia, exhibited 3.5–3.8 times higher maternal HCV rates than metropolitan counties in the United States from 2016–2018 [8]. Despite these increasing rates, evidence remains limited on the determinants of gender differences in HCV and IDU, particularly among young women who inject drugs (YWID) in rural contexts [9]. Understanding the unique experiences of YWID and the factors contributing to their elevated risk for HCV is essential to developing targeted interventions to enhance equitable access to HCV care.

To understand the context of increased IDU and HCV among YWID, one must examine the complex interplay of biological, behavioral, and socio-cultural factors that place them at elevated risk for HCV. Women with opioid dependence report more severe physical and mental health conditions including respiratory, gastrointestinal, and psychiatric conditions, than male counterparts [10]. YWID often rely on their intimate sexual partner for injection assistance, experience challenges in accessing sterile injection equipment, and are more likely to share equipment [11,12]. Women also demonstrate decreased access to housing, employment, and needle exchange services compared to male PWID [13,14]. Additionally, the prevalence of intimate partner violence and transactional sex further exacerbate women’s vulnerability, contributing to injection equipment sharing and HCV risk [9,10,15,16].

Barriers to HCV testing and treatment services pose further challenges for YWID, leading to delayed diagnoses, continued transmission, and suboptimal access to other harm reduction services. Delayed HCV care is often also linked to limited knowledge on HCV transmission, testing, and treatment, along with HCV related misconceptions [17–20]. Women are also less likely to enter HCV care due to financial difficulties, [21] potential ramifications for their children and custody [22,23], and caregiving responsibilities [21,23].

Given the low levels of HCV knowledge, along with the heightened risk of HCV among YWID and their lower likelihood of entering treatment, it is crucial to identify the factors that shape their engagement with HCV care. To better understand these barriers and facilitators, our study applies the Theoretical Domains Framework (TDF), a theory-driven approach designed to explore the determinants of behavior across 14 domains, including knowledge, skills, social influences, and environmental factors [24,25]. This framework provides a comprehensive tool for identifying key gaps in YWID’s HCV knowledge and care engagement, ultimately informing strategies to improve equitable access to HCV testing and treatment. Here, we used the TDF to identify barriers and facilitators to HCV testing and treatment among young women who inject drugs (YWID, ages 18–35) in rural Appalachia Ohio.

## Methods

### Study setting

We conducted in-depth, qualitative interviews to understand HCV testing and treatment experiences of YWID in rural Appalachia Ohio. Our study was conducted in three rural Appalachian counties in southern Ohio: Scioto, Pike, and Jackson. These three Ohio counties have been designated as ‘Distressed’ or ‘At-Risk’ counties based on several economic indicators, including unemployment, per capita income, and poverty rates [26]. The selected counties are within the top 5% of counties in the United States most vulnerable to continued high HCV transmission and the potential for an HIV outbreak among people who inject drugs (PWID) [27].

### Sampling and recruitment

Recruitment for the interviews took place in Ohio counties actively participating in the ongoing National Rural Opioid Initiative (NROI) cohort studies [28]. NROI funding agencies defined rurality according to the “Am I Rural” website (https://www.ruralhealthinfo.org/am-i-rural) from the US Health Resources and Services Administration [28]. Recruitment methods included emails, calls, texts, and in-person at the study office and at local syringe services program (SSP) located in Scioto County. A trained female research assistant was responsible for conducting recruitment and interviews. Participants were eligible if they resided in one of the three counties, were aged 18–35, reported injection drug use in the past 30 days, and recent (past six months) injection with at least one other person. The criteria of injection with at least one other person was included as part of the larger study to capture the influence of injection partnerships on HCV transmission risk. Eligible individuals underwent a consent process, and consenting individuals took part in a one-on-one semi-structured interview with the trained female research assistant. Each interview took place in a private room in the study office to ensure the privacy and safety of participants. Participants were enrolled from August 1^st^ to December 15^th^ of 2023. Participants were given a $35 gift card for completing the interview.

### Data collection

Semi-structured interviews were conducted with 30 YWID in rural Appalachian Ohio. The interview guide covered the following topics: knowledge of HCV and utilization of HCV treatment, including the level of knowledge about the HCV care cascade, sources of HCV information, perceptions related to HCV, trust and openness related to HCV status (self and others), experiences receiving HCV-related care, and access to services. While not directly asked, some participants disclosed their HCV status during the interview. HCV status was not an inclusion or exclusion criterion. All interviews lasted approximately 60 minutes and were audio recorded. After the interviews, participants completed a brief demographic survey. Limited demographic characteristics were collected to protect participant privacy, given the sensitivity of working with a highly distinct and socially interconnected population in close-knit rural communities where confidentiality and anonymity could be threatened.

### Analysis

Interviews were recorded and transcribed verbatim. Three researchers (CW, RM, and AW) coded the interviews in ATLAS.ti using grounded theory to guide coding [29]. CW, an experienced qualitative researcher, supervised the development of the codebook. The three researchers co-coded three interviews to establish inter-coder reliability. Any differences in coding were discussed until all conflicts were resolved. Thematic analysis was employed to derive core themes from coded data. The themes were then mapped onto the TDF domains.

### Ethics

This study was approved by the Ohio State University IRB (IRB#: 2019B0073, Approval date: 02/25/2019). Prior to data collection, research staff obtained written informed consent from participants. All audio files were saved on a password-protected, access-limited server, and any physical copies of the files were kept in a locked, limited-access storage location.

## Results

Twenty-four of the 30 participants reported having been tested for HCV at some point in their lives. Among those who had received HCV testing, 15 reported receiving HCV-positive results, 8 received HCV-negative results, and one did not disclose her status. Two participants reported having completed treatment for HCV and 12 had not pursued treatment at the time of the interview. To protect confidentiality in this small, high-risk sample, more detailed demographic characteristics are not reported.

### Primary TDF domains (Table 1)

**Fig 1:**
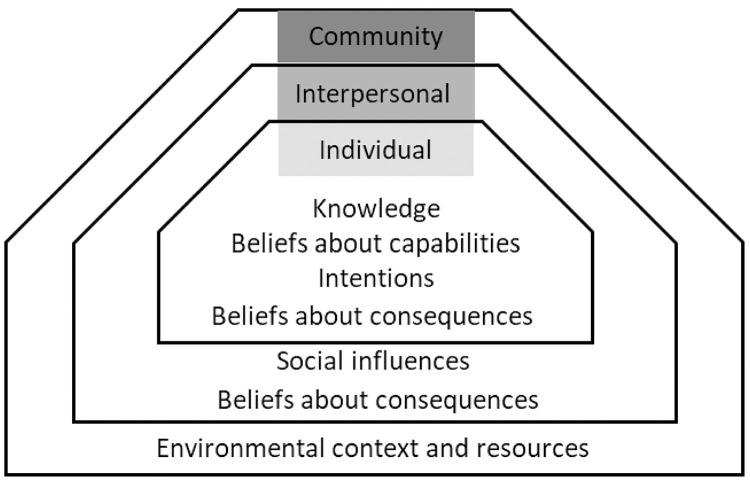
TDF Domains Applied to the Socio-Ecological Model. Fig 1 demonstrates the TDF domains organized into the socio-ecological model.

To demonstrate the spheres of influence for YWID and HCV care, results are presented as levels of a Socio-Ecological Model (SEM) (Fig 1) [30]. The SEM is designed to demonstrate levels of interconnected factors including individual, interpersonal, community, and society. By assigning our results to the levels of the SEM, it allows us to demonstrate the multiple levels of influence on HCV care. One TDF domain, beliefs about consequences, fell across two levels of the SEM. Additionally, not all levels of the SEM were demonstrated through the interviews.

**Table 1:** TDF Domains and Example Quotes.

TDF Domain	Brief Explanation [24]	Example Quote*
**Knowledge**	Knowledge was a facilitator of HCV testing and a barrier to treatment.	“I: How’d you feel after you got your results? P: It scared me to death because I didn’t know nothing about it.”
**Environmental Context and resources**	Transportation to care was the most frequently mentioned barrier to accessing treatment.	“why would you want to take a van or a bus to go get tested? I wouldn’t want to do that...And that’s not very private.”
**Intentions**	Most participants reported being in the pre-contemplation or contemplation stages for accessing treatment.	“I kind of just put it [treatment] on the back burner, like, it’s no big deal, you know what I mean.”
**Beliefs about Capabilities**	A barrier for participants is both feeling incapable of not using injection drugs and feeling incapable of taking a daily treatment pill.	“what’s the point of taking all that medicine when I’m just going to get it [HCV] again?”
**Beliefs about Consequences**	Fear of poor treatment by healthcare providers was a barrier to accessing treatment.	“when you go into seek, like, medical help, when they realize you’re an addict...the attitude and the perspective of me changes, and I’m no longer human, I’m just an addict.”
**Social Influences**	Participants with positive social influences felt motivated to access HCV treatment.	“I’ve kept a job. I’ve kept a house. I’ve paid my bills. And I’ve always kept that [drug use] secret.... I have a family. So I would want to do that [HCV] treatment.”

*I = Interviewer; P = Participant

At the individual level, knowledge, beliefs about capabilities, and intentions emerged as key contributors to HCV care. YWID reported knowing where to access HCV testing and having previously been tested. Compared to testing, the women were more uncertain about HCV treatment, particularly around whether it was “worth it” to access treatment while actively using. While individual knowledge and self-efficacy were individual-level barriers to HCV care, the barriers were often the result of systemic issues. A lack of transparency around HCV treatment restrictions and rapidly changing policies led to outdated or incorrect information being believed by participants related to their ability to access HCV care.

At the interpersonal level, beliefs about consequences and social influences were barriers to HCV treatment and included stigmatization of IDU. Poor treatment of YWID by healthcare providers and administrators led to the participants feeling hesitant to access care for HCV. At the community level, there were two themes that affected HCV care related to the domain of environmental context and resources. One, participants were often tested due to requirements from jails/prisons, treatment centers, employers, or hospitals. Two, lack of transportation was a barrier to HCV care.

### Individual

#### Knowledge.

Knowledge emerged as a facilitator of HCV testing, while lack of knowledge was a barrier to treatment. Most YWID knew where to access HCV testing and were confident in their testing knowledge. Responses to how often HCV testing should occur (n = 25) ranged from weekly (n = 1) to once in their life (n = 1), with the most common response listed as every six months to yearly (n = 9).

While participants knew where to access testing, they lacked comprehensive and accurate information about health outcomes and treatment, contributing to avoidance of testing and heightening the fear of positive results. Participants had a limited understanding of the infectious diseases for which they may be at risk, such as HIV and HCV, conflating a positive result for HCV with HIV. A participant explained, “I: How’d you feel after you got your results? P: It scared me to death because I didn’t know nothing about it. Because in my mind, I’m thinking I brought HIV or something, so I didn’t know.”

Limited knowledge related to HCV treatment was a barrier to accessing treatment. Those who personally or had a family member who received treatment felt more knowledgeable than those who had not. The most mentioned HCV treatment perceptions included the treatment is in pill form (n = 10), and there are serious side effects (n = 9). At the time of the study, few participants were aware of restrictions limited treatment coverage to one time (n = 5). Participants shared outdated guidelines/policies and beliefs about side effects that they perceived to be current. For example, participants shared the belief that active injection drug use would make them ineligible for treatment, a previous requirement that has been removed (n = 3). This outdated understanding of HCV and HCV treatment coverage showed up as participants not wanting to go through treatment if they were still injecting drugs. One participant said, “I just didn’t want to take it and then be like a waste.” Many participants said they did not know anything about HCV treatment, but that HCV was common in their community, “I: Do you know anything about hepatitis C treatment? P: No, I don’t actually. I: Do you know anybody with hepatitis C? P: Well, everybody I know, yeah.”

#### Beliefs about capabilities.

Beliefs about capabilities, specifically perceived behavioral control, emerged as a barrier for participants in both feeling incapable of not injecting drugs and feeling incapable of taking a daily treatment pill. Some participants knew they were not ready to quit using injection drugs and were hesitant to access treatment because they did not want to “waste” the opportunity to be treated while they were actively injecting and at risk of being reinfected. One participant said,

“I: Okay, tell me what you know about hepatitis C treatment. Had you ever had it yourself? P: I have. But I didn’t do, I didn’t take the medicine. I’m just not ready. And what’s the point of taking all that medicine when I’m just going to get it again?”

Other women felt the requirement of the strict treatment routine was too difficult to adhere to, especially when compounded by the feeling of having “wasted” the treatment due to continued drug use,

“What made it hard to stick with the treatment? P: It was the pill I had to take every day at the same time and I’m burnt [out]. I: So just remembering to do that every day was not easy. But you did it for four weeks? P: Yeah. I still was using. I thought man, this is point I’ve wasted this treatment.”

#### Intentions.

Intentions emerged as a barrier with most participants reporting being in the precontemplation or contemplation stages for accessing treatment. One participant stated, “I kind of just put it [treatment] on the back burner, like, it’s no big deal, you know what I mean.” Another participant stated, “I feel like, maybe once I got sober...then I’d probably care more about my health.”

### Individual/Interpersonal

#### Beliefs about consequences.

Beliefs about consequences fell across two SEM domains, as perceived consequences of HCV treatment fell into the individual domain (fear of side effects) and interpersonal domain (fear of stigma from care providers).

**Individual.** Beliefs about consequences, specifically perceived treatment side effects, influenced whether participants wanted to access treatment. Participants frequently listed strong side effects as a reason they may not continue treatment, “I: Can you think of something that would keep you from continuing treatment if you were taking it? P: Maybe if there was side effects that were hard to deal with.”

**Interpersonal.** Beliefs about consequences also showed up as perceived stigma by healthcare providers, a barrier to accessing HCV care. Participants who felt stigmatized by care providers did not want to access care,

“...when you go into seek, like, medical help, when they realize you’re an addict...you can see the look on their face and the way they talk to you and their demeanor can change. I can cover my track marks, I can get dressed every day and look a certain way, but once they find out I am who I am and what I am, the attitude and the perspective of me changes, and I’m no longer human, I’m just an addict.”

This domain emerged as outcome expectancies as participants often reported feeling aware of positive HCV status prior to testing. These participants said getting the positive result was like a “reality check,” with one participant expressing, “I knew that I had it, but it’s still kind of like damn.”

### Interpersonal

#### Social influences.

Participants with jobs and families unaware of their injection drug use reported they would proceed with treatment regardless of side effects, “I’ve kept a job. I’ve kept a house. I’ve paid my bills. And I’ve always kept that secret.... I have a family. So I would want to do that treatment.”

The stigmatization by healthcare providers also fell within the social influence domain. Stigmatization was a social influence as it related to individual providers, rather than the healthcare system as a structural unit. YWID with positive experiences with providers noted they would happily return to the same care location for testing or treatment, lending to continuity in care, “I: How’d the staff treat you when you went [to the health department]? P: They treated me good. I: They treated you good? So is that some place you would go back to? P: Yeah.”

### Community

#### Environmental context and resources.

Environmental context and resources significantly influenced participants’ behavior. Many participants who reported being tested for HCV received their HCV test due to their environmental circumstances. Of those who disclosed why they were tested (n = 17), nearly half received mandatory testing while in jail/prison, at a treatment center, during a hiring process, or during hospitalization (n = 8). Participants’ feelings about mandatory testing were shaped by their care quality and ranged from negative to neutral.

Transportation to care was the most frequently mentioned barrier to accessing treatment. Public transportation was not widely available in the rural region and SSPs were often located far from where participants resided. Participants lacked personal vehicles and did not want to use ride sharing due to the lack of privacy. “Why would you want to take a van or a bus to go get tested? I wouldn’t want to do that...And that’s not very private.”

### Selection of TDF domains

The above topics could have been organized into other TDF domains. The fear of being judged when seeking HCV care was categorized under the “beliefs about consequences” domain, though it could also align with the “emotion” or “reinforcement” domains. This placement was made because, rather than solely reflecting negative feelings or consequences associated with stigma, participants demonstrated an acceptance of the anticipated outcomes tied to their identity as PWIDs when engaging with healthcare services.

## Discussion

YWID face pervasive barriers to accessing HCV care in rural Appalachia, as revealed through the application of the TDF to understand their experiences. These barriers span multiple TDF domains, including knowledge, beliefs about consequences, and environmental context and resources, offering critical and novel insights into the complex interplay of misinformation, stigma, and resource constraints that limit care engagement. Our findings underscore the need for multi-level implementation strategies that address misconceptions about HCV care, stigma, and transportation barriers. Specifically, targeted interventions integrating education, stigma reduction, and motivational support while leveraging social influences present a significant opportunity to enhance HCV care engagement among YWID. These findings align with prior research, which emphasizes the intersection of stigma, substance use, and structural barriers in limiting access to healthcare services for PWID [31–33]. Importantly, our study extends existing evidence by centering on the unique experiences of young women in rural settings, a group often underrepresented in implementation science research.

The Theoretical Domains Framework has been used to identify facilitators and barriers and identify implementation strategies for addressing infectious diseases and substance use [34,35]. Recently, the TDF has been implemented in both provider and patient-facing studies about HCV and PWID [36,37]. In studies using the TDF to discuss HCV and injection drug use, authors found similar themes to those in our study. Participants in our study and a 2024 study with PWIDs in Montreal Canada [36] discussed the fear of discrimination and poor treatment by health care providers as barriers to testing and treatment. The authors explained the stigma felt by participants was intersectional, relating to both their statuses as PWIDs and as HCV positive. Fontaine et al. explained that participants felt the testing/treatment process was stigmatizing due to the extensive questioning of lifestyle practices (belief about consequences). To address stigma, healthcare providers need sensitivity training for caring for those with substance use disorders (SUDS) [38]. Programs demonstrating significantly decreased perceptions of negative stereotypes [39] and improved attitudes by providers toward people who use substances [40], as well improvements in the quality of the provider-client interactions [41] all centered putting clinicians in contact with people with SUDS.

In our study, YWID were aware of where to access HCV testing but lacked accurate information about health outcomes and treatment. This aligns with a 2019 study in rural Kentucky with young adults who inject drugs, which found participants lacked knowledge about HCV transmission risks [42]. Rapidly changing policies lead to outdated beliefs and misconceptions about eligibility and treatment limitations. Lack of awareness around treatment improvements fuels fears about treatment side effects.

Medicaid previously enforced many behavioral restrictions (Table 2) for covering HCV treatment, including abstinence from drugs or alcohol [48]. Recent policy shifts have removed many behavioral restrictions for treatment coverage, such as abstinence from drug or alcohol use, though requirements still vary by state. Ohio no longer imposes restrictions based on substance use as of 2020, about three years prior to this study, though some participants believed this restriction was still in place. Ohio does still requires prior authorization, adherence counseling, and specific documentation for therapy approval [49]. At the time of the interviews, the restriction of one-time treatment coverage was still in place, but this changed as of August of 2023. Only five participants in our study mentioned this restriction, demonstrating a lack of familiarity with existing limitations for HCV treatment. While changes are mandated at the state level, removal of restrictions are growing, with significant improvements in care access across the U.S. As of September 2024, no states mandate abstinence, but some still impose retreatment restrictions or required providers to address substance use [47]. Thirteen states have retreatment restrictions based on adherence, substance use, or SVR12 documentation [50]. For comparison, in 2017, 27 states required patients to abstain from drug use from between 1–12 months prior to treatment [51] and until June of 2022, 23 states had retreatment restrictions [52]. While these changes improve care access, the evolving regulations create challenges for YWID and healthcare providers in maintaining up-to-date knowledge, often leading to misinformation about treatment access. To optimize the benefits of such changes, information needs to be more widely and quickly disseminated among healthcare providers and patients. Addressing these gaps through targeted, evidence-based education is vital for empowering YWID to seek care and engage in HCV treatment.

**Table 2:** Medicaid Restrictions and Ohio Policies.

Restriction^a^	Definition^a^	Current Ohio Policy as of February 2025	Year restriction removed or changed in Ohio
Prior authorization	Requiring prescribers to obtain advance approval before insurance will cover HCV treatment	Prior authorization is required for all HCV treatment regimens^b^	While this restriction is still in place, in 2020, Ohio implemented a Unified Preferred Drug List that encompasses all Medicaid enrollees, which streamlined treatment authorization^d^
Fibrosis restrictions	Based on a patient’s degree of liver damage or fibrosis level	Ohio Medicaid does not impose fibrosis restrictions^b^	Restriction was present in 2017 and removed by 2019^e^
Substance use restrictions	Based on a patient’s drug or alcohol use	Ohio Medicaid does not impose substance use restrictions^b^	Restriction removed as of 5/1/2020^d^
Prescriber restrictions	Based on a prescriber’s specialty or expertise	Ohio Medicaid does not impose prescriber restrictions^b^	Removed between January and June of 2022^e^
Retreatment restrictions	Based on whether a patient has previously received treatment	Ohio Medicaid does not restrict access to retreatment^b^	Removed between August 2023 and February 2024^e^
Access in managed care	Assesses parity between a state’s fee-for-service program and contracted managed care organizations	All MCOs impose the same requirements as FFS according to the uniformed preferred drug list^c^	Additional restrictions or lack of transparency in managed care removed between June 2022 and February 2023^e^
Additional restrictions	Additional restrictions to treatment that are less common but may pose severe barriers to care	Ohio Medicaid imposes additional restrictions as follows:^b^ • HCV RNA must be collected within 180 days of application for therapy. • Documentation of genotype. • Documentation of adherence counseling	Not previously or consistently reported^e^

^a^[43]

^b^[44]

^c^[45]

^d^[46]

^e^[47]

Targeted educational campaigns can address common misconceptions, such as outdated beliefs about treatment eligibility and coverage [53,54]. These campaigns could be disseminated through familiar and trusted community spaces, including SSPs, health departments, and primary care clinics [55]. Additionally, a 2017 publication examining HCV status disclosure among PWID in rural Appalachia found the majority (69.1%) of participants disclosed their status to a friend, family member, or partner [56], demonstrating an opportunity for HCV educational campaigns that extend beyond PWID.. Educational efforts should not make assumptions about existing knowledge and explicitly address gaps in understanding about HCV transmission, testing, and treatment options, including recent changes to Medicaid restrictions. Changes to previous policies, such as changes to HCV health insurance coverage for those still actively injecting, should be central in awareness messaging to ensure updated guidelines are communicated.

YWID in our study described how stigma—both perceived and enacted—discouraged engagement with HCV care services, which is consistent with prior evidence. Within the TDF domain of beliefs about consequences, YWID expressed fear of judgment from healthcare providers. In a recent study in Montreal, Canada, both PWID and harm reduction providers identified fear of discrimination and poor treatment by healthcare providers as significant barriers to HCV testing and treatment [36]. This stigma was intersectional, stemming from participants’ dual identity as PWID and as individuals at risk for or living with HCV [36]. Additionally, the HCV testing and treatment procedures themselves often reinforced stigma, as they include extensive questioning about behaviors [36]. Being young and a woman adds further layers of intersectionality to the stigma experienced by YWID, as societal stereotypes around youth, gender, and substance use compound their fear of judgment and discrimination in healthcare settings. These intersecting identities can amplify vulnerability, with young women often facing additional scrutiny and judgment related to their substance use, reproductive health, and perceived roles in society, further discouraging engagement with HCV care. Addressing this stigma could include educational and sensitivity training for healthcare providers and administrative staff to better understand the unique challenges faced by YWID [57]. This should also include teaching clinicians strategies to better equip their patients to adhere to treatment (e.g., methods to help remember to take a daily medication, managing side effects, and how to store medication if housing is inconsistent). Gender-specific training may help reduce the fear of judgment that often discourages YWID from engaging with HCV care services.

YWID noted transportation barriers, a critical component of the environmental context, hindering access to HCV care, which other studies have consistently noted as a primary barrier for PWIDs to access care [58–61]. Studies have also used the TDF to identify barriers and facilitators to HCV care from the physician’s perspective. The findings in these studies mirror many of the concerns voiced by our participants. Clinicians note the need to de-centralize HCV testing, making it community-embedded and thus more accessible [37]. Expanding mobile care units at SSPs and areas with high concentrations of YWID, along with low-barrier private transportation options such as voucher programs or discreet shuttles, could improve healthcare access by reducing reliance on public transit and ride-share services [62]. However, addressing stigma and privacy concerns, including fears of being seen at clinics or law enforcement targeting SSPs, is essential for successful implementation. This aligns with prior evidence using the TDF to examine HCV care barriers, including those from the perspective of healthcare providers. Clinicians have emphasized the need to decentralize HCV testing, embedding it in community settings to enhance accessibility [63]. Harm reduction providers have expanded on this, highlighting the absence of permanent healthcare providers at SSPs as a notable barrier to care [36]. As a result, studies are underway expanding mobile harm reduction access through vans or pharmacies [64–67]. Addressing these gaps by integrating mobile or community-embedded care services offers a promising strategy for reducing logistical barriers and improving treatment uptake among YWID.

It is important to note, most participants were recruited from the most heavily populated of the three counties, with challenges arising in reaching YWID from more rural and sparsely populated areas. As a result, the findings may not fully reflect the experiences of women in less populated regions, who may face distinct barriers to accessing HCV and harm reduction services. Additionally, the sample predominantly consisted of white participants, reflecting the demographics of the study area. Given the sensitive nature of this research and the close-knit nature of rural communities, ensuring confidentiality and anonymity was a priority, which limited the extent to which detailed demographic characteristics could be captured. Future research should aim to include a more racially and ethnically diverse sample of rural people who use drugs, as well as consider other intersecting characteristics, such as gender, sexuality, and disability, to better capture diverse experiences with HCV care and barriers to engagement. These limitations highlight the ongoing challenges of studying populations that have been socially and economically marginalized in rural, geographically isolated areas like Appalachia.

## Conclusions

YWID in rural Appalachia face numerous barriers to HCV care, spanning multiple TDF domains. Gaps in knowledge about HCV treatment, compounded by gendered stigma and logistical challenges demonstrated in our results, highlight the need for interventions specifically designed to address YWID lived experiences. Educational campaigns tailored to address the updated treatment guidelines and health concerns of young women will be essential for improving their understanding of HCV care. Stigma-reduction training for healthcare providers may also be crucial, particularly training which addresses gender-related and substance use-related biases described by our participants. Additionally, decentralized care models, which bring services closer to where young women live and reduce the need for extensive transportation, represent a promising step forward in improving access to HCV care. Future implementation science research should focus on evaluating these approaches, ensuring they are responsive to the intersectional and gender-specific challenges faced by YWID. Such efforts will be essential for building equitable and effective HCV care systems that prioritize the unique needs of YWID in Appalachia and similar rural settings. Studies have demonstrated improved HCV knowledge and individualized HCV education result in increased interest in receiving HCV treatment [17,68–70]. The significant gaps in knowledge among YWID related to HCV treatment demonstrate a need for education about HCV treatment and the dispelling of myths. The hesitancy of YWID with HCV to access treatment may require reducing care barriers such as interventions targeted at care providers to reduce stigmatization of PWIDs and being clear about the side effects of HCV treatment and how to manage them [57]. Additional research is needed to identify strategies to address knowledge, stigma among health care providers, and access to services.

## Abbreviations

HCV: Hepatitis C Virus
IDU: Injection Drug Use
PWID: People Who Inject Drugs
SEM: Socio-Ecological Model
SSP: Syringe Services Program
SUDs: Substance Use Disorders
TDF: Theoretical Domains Framework
YWID: Young Women who Inject Drugs
